# Integrated meta-omics reveals new ruminal microbial features associated with feed efficiency in dairy cattle

**DOI:** 10.1186/s40168-022-01228-9

**Published:** 2022-02-16

**Authors:** Ming-Yuan Xue, Yun-Yi Xie, Yifan Zhong, Xiao-Jiao Ma, Hui-Zeng Sun, Jian-Xin Liu

**Affiliations:** 1 Institute of Dairy Science, College of Animal Sciences, Zhejiang University, Hangzhou, 310058 China; 2 Ministry of Education Key Laboratory of Molecular Animal Nutrition, Zhejiang University, Hangzhou, 310058 China; 3 Ministry of Education Innovation Team of Development and Function of Animal Digestive System, Zhejiang University, Hangzhou, 310058 China

**Keywords:** Dairy cattle, Feed efficiency, Metabolomics, Metagenomics, Metatranscriptomics, Rumen microbiome

## Abstract

**Background:**

As the global population continues to grow, competition for resources between humans and livestock has been intensifying. Increasing milk protein production and improving feed efficiency are becoming increasingly important to meet the demand for high-quality dairy protein. In a previous study, we found that milk protein yield in dairy cows was associated with the rumen microbiome. The objective of this study was to elucidate the potential microbial features that underpins feed efficiency in dairy cows using metagenomics, metatranscriptomics, and metabolomics.

**Results:**

Comparison of metagenomic and metatranscriptomic data revealed that the latter was a better approach to uncover the associations between rumen microbial functions and host performance. Co-occurrence network analysis of the rumen microbiome revealed differential microbial interaction patterns between the animals with different feed efficiency, with high-efficiency animals having more and stronger associations than low-efficiency animals. In the rumen of high-efficiency animals, *Selenomonas* and members of the *Succinivibrionaceae* family positively interacted with each other, functioning as keystone members due to their essential ecological functions and active carbohydrate metabolic functions. At the metabolic level, analysis using random forest machine learning suggested that six ruminal metabolites (all derived from carbohydrates) could be used as metabolic markers that can potentially differentiate efficient and inefficient microbiomes, with an accuracy of prediction of 95.06%.

**Conclusions:**

The results of the current study provided new insights into the new ruminal microbial features associated with feed efficiency in dairy cows, which may improve the ability to select animals for better performance in the dairy industry. The fundamental knowledge will also inform future interventions to improve feed efficiency in dairy cows.

Video Abstract.

**Supplementary Information:**

The online version contains supplementary material available at 10.1186/s40168-022-01228-9.

## Background

Dairy cattle are an important source of high-quality animal protein for human consumption. Along with the increasing global population and competition for resources between humans and livestock, meeting the demand for high-quality dairy protein has become a global food security concern [1]. Hence, increasing milk protein production and improving feed efficiency have become the most coveted goals in the dairy industry [2]. Feed efficiency in ruminants is determined largely by the ability of the rumen microbiome to convert potentially digestible feedstuffs into metabolizable nutrients [3]. The ability to digest various plant masses differs among individual cows and is largely dependent on the diverse microbiomes in their digestive tracts, particularly in the rumen [4, 5]. In recent years, the advancement of understanding of the associations between the rumen microbiome and feed efficiency has led to novel strategies to select high-efficiency animals based on their rumen microbiomes [6–9]. The rumen microbiome is responsible for the deconstruction and fermentation of feed plant fibers and converts plant materials to volatile fatty acids (VFAs), which serve as the main energy source for ruminants [10]. Rumen microbes are also an important protein source for ruminants, as they are subsequently digested in the small intestine [11]. Therefore, understanding the microbe-dependent mechanisms underlying feed efficiency microbial features associated with feed efficiency in dairy cows is of great importance.

In the past decade, compositional variation in the rumen microbiomes and their effects on host feed efficiency and methane emissions have been investigated using PCR-denaturing gradient gel electrophoresis (DGGE) [6, 7, 12] and meta-taxonomics [8, 13, 14]. The functional potential of the rumen microbiome and its relationship with animal performance have also been extensively examined using metagenomics and metatranscriptomics [15, 16]. As expected, a recent study comparing the rumen metagenomes and metatranscriptomes of beef cattle suggested that the metatranscriptomes can closely represent the active functions of the microbiomes [17]. However, the metabolic functions of the rumen microbiome are better conserved than the taxonomic compositions [16, 18, 19], and microbial metabolites make greater contributions to host phenotypes than taxonomic compositions [20]. These findings suggest that the rumen metabolome should also be considered when associating the rumen microbiome with host feed efficiency. However, few studies have focused on the metabolic variation in the rumen microbiome and its relationship with host feed efficiency, especially in dairy cows [21]. This knowledge gap hinders the elucidation of the rumen microbiome-dependent mechanisms underpinning feed efficiency in dairy cows and other ruminants.

To date, knowledge of the rumen metatranscriptomic and metabolomic profiles of dairy cattle remains limited, especially with respect to their linkages with host phenotypes. Therefore, integration of the compositional profiles and active functions, together with the metabolites of the rumen microbiome, is needed to elucidate the microbiome-dependent mechanisms underlying the feed efficiency of dairy cattle. In the current study, multiple meta-omics approaches, including metagenomics, metatranscriptomics, and metabolomics of the rumen contents, were applied and integrated to address the following questions: (1) How do microbes interact with each other and function in the rumen of cows with different feed efficiencies? (2) Can some of the rumen microbial features be used as predictive markers for feed efficiency and potentially used in selecting cows with high feed efficiency? The answers to these questions will advance our knowledge regarding the microbial features associated with host feed efficiency and help improve selecting cows for better performance in the dairy industry.

## Methods

### Animals and samples

**Fig. 1:**
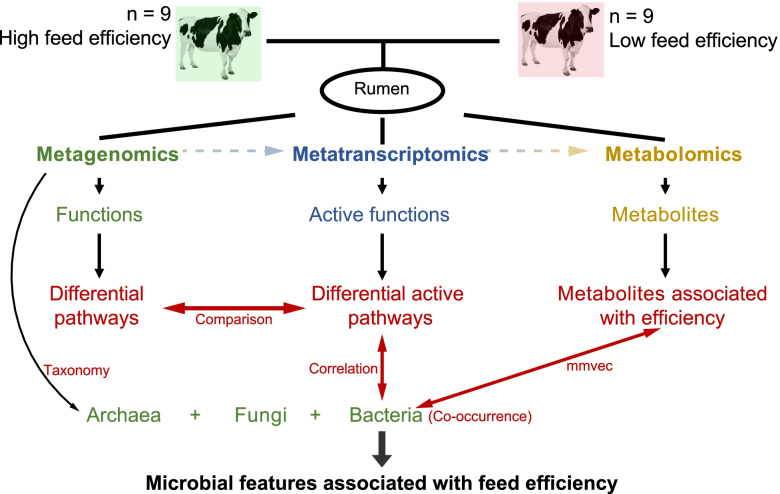
Workflow of the integrated rumen metagenomes, metatranscriptomes, and metabolomes

The experimental protocol was approved by the Animal Care Committee of Zhejiang University (Hangzhou, China). The experimental design is presented in Fig. 1. A total of 60 mid-lactating Holstein dairy cows (parity = 2.48 ± 0.62) were selected from a herd of 323 dairy cattle (Hangzhou, China) and used for measurement of feed intake. The 60 cows were fed a total mixed ration that was formulated to produce 35 kg of milk per day with 3.25% milk protein [22]. The feed intake data were recorded using automatic weighting troughs (Roughage Intake Control System, Marknesse, The Netherlands) to calculate the dry matter intake (DMI) as described previously [22]. Rumen content samples were collected using oral stomach tubes [23] before morning feeding and preserved at −80°C until analysis in the present study. The feed conversion rate (FCR) of the 60 cows was calculated using their DMI and milk (3.5% fat-corrected milk) yield data, and 18 animals were then selected and divided into two groups: high-efficiency (HiEf) cows and low-efficiency (LoEf) cows (Fig. 1). Power calculations revealed that the sample size enabled 99.67% power and a type 1 error of 5% (effect size = 0.78, Cohen’s d = 2.53) based on a *t* test of FCR.

### Metagenomic sequencing and data processing

The methods for DNA extraction from the rumen content samples, metagenomic sequencing, and data processing were the same as reported previously [20]. In brief, total genomic DNA was extracted from the rumen contents using repeated bead-beating plus column purification [24]. Individual metagenome libraries were constructed using the TruSeq DNA PCR-Free Library Prep Kit (Illumina, San Diego, CA, USA). The metagenome libraries were sequenced (2 × 150 paired-end) on the Illumina HiSeq 3000 platform at Majorbio Bioinformatics Technology Co., Ltd. (Shanghai, China).

Quality control of the metagenomic sequence reads was performed using Sickle (version 1.33, https://github.com/najoshi/sickle). The quality-filtered reads were then aligned to the bovine genome (bosTau8 3.7, DOI: 10.18129/B9.bioc.BSgenome.Btaurus.UCSC.bosTau8) using BWA v0.7.1 (http://bio-bwa.sourceforge.net) to filter out the host DNA sequences [25], and the remaining reads were de novo assembled using Megahit v1.1.2 [26]. The assembled contigs were subjected to prediction of open reading frames (ORF) using MetaGene v0.3.38 [27]. Nonredundant contigs were identified using CD-HIT at 95% sequence identity and 90% coverage [28]. The original sequencing reads were mapped to the predicted genes to estimate their abundance using SOAPaligner v2.21 [29]. The contigs were taxonomically assigned using DIAMOND [30] against the RefSeq database [31]. Taxonomic profiles were examined at the domain, phylum, genus, and species levels, and relative abundances at those taxonomic ranks were calculated using HUMAnN v2.0 (https://huttenhower.sph.harvard.edu/humann2/). For potential functional profiles, the contigs were annotated using DIAMOND against the KEGG database (http://www.genome.jp/kegg/) with an *E* value of 1e−5. Abundances of KEGG Orthology (KO), pathways, and KEGG enzymes were normalized into counts per million reads (CPM) for downstream analysis. Exogenous pathways were selected based on a previous study and were excluded from downstream analysis [19].

### Metatranscriptomic sequencing and data processing

The microbial RNA was extracted from the rumen content samples as described previously [32], and RNA samples with an RNA integrity number (RIN) > 7.0 were used in generating metatranscriptome libraries after mRNA enrichment [33]. Metatranscriptomic sequencing (2 × 150 paired-end) was performed on the Illumina HiSeq 3000 platform at Majorbio Bioinformatics Technology Co., Ltd. (Shanghai, China).

The RNA-Seq reads were subjected to quality filtering using Trimmomatic (version 0.39) [34]. TopHat (version 2.1.1) was used to remove host sequences by comparison against the bovine genome [35]. De novo assembly was performed using Meta-Velvet (version 1.2.01) [36]. Functional annotation was performed using UBLAST that is implemented in USEARCH (version 11) [37] against the KEGG database, with an *E* value of 1e−5. The RNA-seq reads were mapped to the assembled sequences, and the mapping outputs were further converted to count files using HUMAnN v2.0 (https://huttenhower.sph.harvard.edu/humann2/). Abundances of KEGG Orthology (KO), pathways, and KEGG enzymes were normalized into CPM for downstream analysis, and exogenous pathways were excluded [19]. Taxonomic profiles were analyzed using the MG-RAST pipeline against the RefSeq database [38] and the Simple Annotation of Metatranscriptomes by Sequence Analysis (SAMSA) software package [39]. Taxonomic profiles were examined at the domain, phylum, genus, and species levels, and relative abundances were calculated for each taxonomic rank.

### Metabolomic analysis and data processing

The rumen metabolome was analyzed using gas chromatography (Agilent Technologies, Santa Clara, CA, USA) coupled to Pegasus HT time-of-flight/mass spectrometry (GC-TOF-MS; LECO Corporation, St. Joseph, MI, USA). Data processing was performed as previously reported [20]. The Chroma TOF 4.3X software (LECO Corporation, St. Joseph, MI, USA) and the LECO-Fiehn Rtx5 database [40] were used for raw data filtering and processing. Metabolite peaks that were present in > 50% of the samples or with a relative standard deviation < 30% and with a similarity value > 200 were used in downstream analysis. Metabolites were correlated with the FCR phenotypes using Spearman correlation to select FCR-associated rumen metabolites.

### Bioinformatics and statistical analysis

Co-occurrence among the bacterial taxa was analyzed using the SparCC program with the default settings [41]. Spearman correlation analysis was performed to associate microbial taxa with the transcriptionally active functions (active functions hereafter). Only the genus-level bacterial taxa with a relative abundance > 0.1% and prevalence > 50% were used in the co-occurrence and correlation analysis, and only those with a correlation coefficient of > 0.5 or < −0.5 and a *P* value of < 0.05 were used in co-occurrence network analysis. Networks were visualized using Cytoscape (Version 3.2.1, http://www.cytoscape.org). The hubs of the microbes in the networks were calculated using the “CytoHubba” function in the Cytoscape software based on the Maximal Clique Centrality (MCC) method (https://apps.cytoscape.org/apps/cytohubba).

The randomForest package in R was used for the random forest analysis [42], with the rumen metabolites being used as the inputs of the random forest model. The mean decrease accuracy (MDA) score, which reflects the importance of metabolites in the model, was given to each metabolite based on the increase in error caused by removing that metabolite from the predictors. The best predictive metabolites were identified based on the maximum area under the curve (AUC) using the UC-RF algorithm. A 99-fold cross-validation scheme was applied for further evaluation of the model using the rfUtilities package in R (Version2.1-5, https://cran.r-project.org/web/packages/rfUtilities/index.html).

To reveal the relationship between the rumen microbes and metabolites detected, microbe-metabolite vectors (mmvec), which predict the entire metabolite abundance profile from a single microbial sequence, were applied [43], and the resultant mmvec neural network was used to learn the co-occurrence probabilities between microbes and metabolites. The interactions between microbes and metabolites were ranked and visualized through the standard dimensionality reduction interface that is implemented as a plugin in QIIME2 (Version 2020.8) [44].

Non-metric multi-dimensional scaling (NMDS) analysis was performed based on Bray-Curtis dissimilarity using the Vegan package in R (https://www.r-project.org). Analysis of Similarities (ANOSIM) was performed based on the Bray-Curtis dissimilarity (999 permutations) using the Vegan package in R. Statistical analyses were performed using R. Phenotypic data were compared between the two groups using a *t* test. The relative abundances of microbial taxa and CPM of metagenomic and metatranscriptomic functions were compared between the two groups using the Wilcoxon rank-sum test. The *P* values were adjusted by the false discovery rate (using the BH method of the stats package in R) [45]. Only the taxa with a relative abundance > 0.1% and only the functions with CPM > 5 were compared between the two groups of cows. Fold changes of CPM of microbial functions were presented. Differences with a *P* value < 0.05 were considered significant.

## Results

### Animal phenotypes, rumen fermentation characteristics, and metagenomic and metatranscriptomic data statistics

**Fig. 2:**
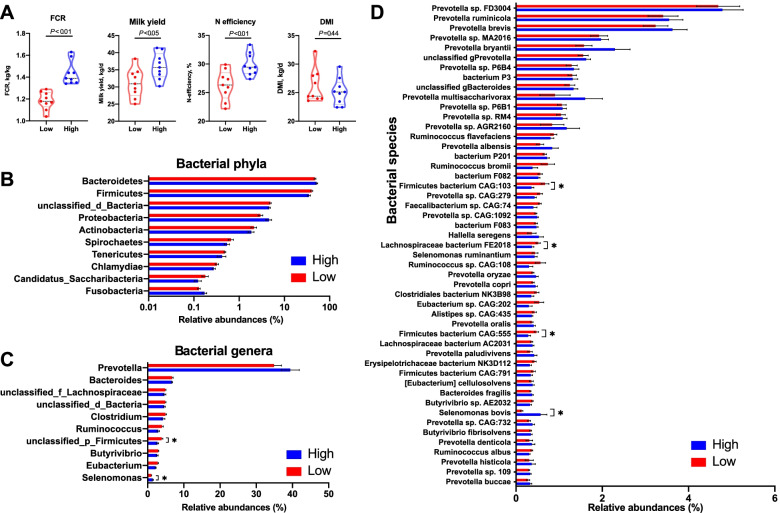
Comparison of phenotypic data and rumen bacterial taxa identified in the metagenomes between cows with different feed efficiencies. Feed conversion rate (FCR), milk yield, nitrogen (N) efficiency, and dry matter intake (DMI) were compared using a *t* test (**A**). The 10 most abundant bacterial phyla (**B**), 10 most abundant bacterial genera (**C**), and 50 most abundant bacterial species (**D**). The Wilcoxon rank-sum test was used for mean comparison. *P < 0.05

Dry matter intake was similar between the HiEf and the LoEf cows (*P* = 0.444), but milk yield, FCR, and *N* efficiency were higher (*P* < 0.05) in the HiEf cows than in the LoEf cows (Fig. 2A and Supplementary Table S1). The HiEf cows also had a higher (*P* = 0.002) energy-corrected milk (ECM): DMI ratio than the LoEf cows (Supplementary Table S1). The two groups of cows did not differ in milk urea nitrogen or any of the determined rumen fermentation characteristics.

A total of 138.52 Gb of data were obtained from the metagenomic sequencing, with 7.70 ± 0.46 Gb per sample (Supplementary Table S2). A total of 137.16 Gb of data were retained after quality filtering and removing host DNA sequences. A total of 11,935,765 contigs were generated from de novo assembly (663,098 ± 145,797 per sample, N50 length of 778 ± 124).

Metatranscriptomic sequencing generated a total of 162.34 Gb of data (9.02 ± 1.25 Gb per sample). A total of 127.58 Gb of data were remained after quality control, mRNA enrichment, and filtering out host RNA reads (Supplementary Table S2). After de novo assembly, an average of 63,989 ± 22,058 contigs per sample were obtained (N50 length of 640 ± 122).

### Rumen microbiome composition as determined by metagenomics and metatranscriptomics

The overall microbial NMDS based on metagenomic data showed two clusters between the two groups (stress = 0.101, ANOSIM *R* = 0.325, *P* < 0.05, Supplementary Figure S1A). From the metagenomic sequences of bacteria (26,591,712 ± 1,926,636 sequences per sample), a total of 62 phyla, 1339 genera, and 5362 species of bacteria were identified (data not shown), of which 12 phyla, 24 genera, and 20 species were considered as predominant bacterial taxa (each with a relative abundance > 0.5% in at least one sample and a prevalence > 20%, Supplementary Table S3). None of these predominant bacterial phyla differed in relative abundance between the two cow groups (Fig. 2B). At the genus level, an unclassified *Firmicutes* genus was more abundant (*P* < 0.05) in the LoEf cows than in the HiEf cows, while *Selenomonas* showed the opposite trend (Fig. 2C). At the species level, three uncultured species of *Firmicutes* had a higher abundance (*P* < 0.05) in the LoEf cows than in the HiEf cows, but *Selenomonas bovis* showed the opposite abundance trend (Fig. 2D).

The archaeal metagenomic sequences (391,764 ± 145,445 per sample) were taxonomically classified to a total of 6 phyla, 49 genera, and 117 species of archaea (data not shown), of which one phylum, 6 genera, and 13 species were predominant (as defined above for the predominant bacterial taxa, Supplementary Table S3). At the phylum level, only one low-abundance archaeal phylum (i.e., candidate *Bathyarchaeota*) was more abundant (*P* < 0.05) in the rumen of the HiEf cows than the LoEf cows (Supplementary Table S3). *Methanobrevibacter*, which was the most abundant genus of archaea, showed a lower abundance (*P* < 0.05) in the HiEf cows than in the LoEf cows (Supplementary Table S3). No species-level archaeal taxa differed in relative abundance between the two cow groups (Supplementary Table S3). An average of 582,310 ± 526,904 fungal metagenomic sequences were generated per sample. These sequences were taxonomically assigned to 10 phyla, 120 genera, and 146 species (data not shown), with 20 genera and 15 species being predominant (as defined above for the predominant bacterial taxa, Supplementary Table S3). None of the detected fungal taxa differed in relative abundance between the two cow groups (Supplementary Table S3).

The overall microbial NMDS based on metatranscriptomic data showed clear separations between the two groups (stress = 0.072, ANOSIM *R* = 0.410, *P* < 0.05, Supplementary Figure S1B). Based on metatranscriptomic sequencing data, an average of 17,291,314 ± 823,431, 182,657 ± 105,195, and 361,030 ± 221,841 sequences were generated per sample for bacteria, archaea, and fungi, respectively. Alpha diversity indexes of microbiome were compared based on metatranscriptome data, only archaea had a higher Shannon index in the HiEf cows than in the LoEf cows (*P* < 0.05, Supplementary Figure S2). None of the predominant bacterial phyla showed any difference (*P* > 0.05) between the two cow groups (Supplementary Table S4). At the genus level, *Selenomonas* was more abundant (adjusted *P* < 0.05) in the HiEf cows than in the LoEf cows, while the opposite was true for *Oscillibacter* (Supplementary Table S4). At the species level, five taxa differed in relative abundance between the two cow groups, with *Selenomonas bovis* being more abundant in the HiEf cows than in the LoEf cows, and four unclassified species having higher abundances in the LoEf cows than in the HiEf cows, including two species of *Firmicutes* and one taxon each of *Prevotella* and *Lachnospiraceae* (adjusted *P* < 0.05, Supplementary Table S4). Of the identified archaeal taxa, only the genus *Methanobrevibacter* had different relative abundance between the two cow groups (more abundant in the rumen of LoEf cows, adjusted *P* < 0.05). None of the fungal taxa were different between the two groups (Supplementary Table S4).

### Rumen microbial functions as determined by metagenomics and metatranscriptomics

**Fig. 3:**
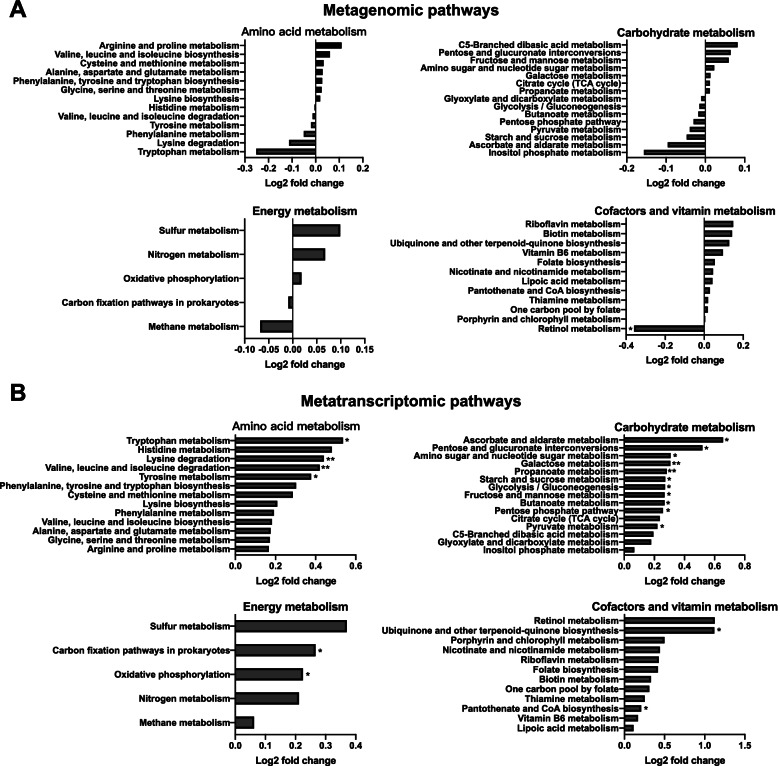
Fold changes of metabolic pathways identified in the metagenomes and metatranscriptomes of the cows with high and low feed efficiencies. **A** Pathways identified in the metagenomes and **B** pathways identified in the metatranscriptomes. The Wilcoxon rank-sum test was used for mean comparison. *P < 0.05

The metagenomic sequences were mapped to a total of 249 KEGG level-3 pathways. After excluding exogenous pathways and pathways with low abundance and prevalence (CPM < 5 and prevalence < 20%), 218 KEGG level-3 pathways remained (Supplementary Table S5). These pathways were assigned to four level-1 categories, including “Metabolism” (71.04%), “Genetic Information Processing” (15.39%), “Environmental Information Processing” (6.87%), and “Cellular Processes” (6.70%). Comparison of the 20 most abundant microbial pathways between the two groups showed no difference (*P* > 0.05) (Supplementary Figure S3). We further selected the key pathways involved in amino acid metabolism, carbohydrate metabolism, energy metabolism, and cofactors and vitamin metabolism. Only one pathway, “Retinol Metabolism,” differed between the two cow groups, being less abundant (*P* < 0.05) in the rumen of the HiEf cows (Fig. 3A). The overall functional NMDS based on metagenomic data showed two clusters between the two groups (stress = 0.063, ANOSIM *R* = 0.367, *P* < 0.05, Supplementary Figure S1C).

In the rumen metatranscriptomes, 358 KEGG level-3 pathways were identified. After excluding exogenous pathways and pathways with low abundances and prevalence (as defined above for the pathways identified in the metagenomes), 193 KEGG level-3 pathways were remained (Supplementary Table S6). These microbial pathways were assigned four level-1 categories, including “Metabolism” (49.22%), “Genetic Information Processing” (29.86%), “Environmental Information Processing” (13.07%), and “Cellular Processes” (7.84%). The overall functional NMDS based on metatranscriptomic data showed clear separations between the two groups (stress = 0.051, ANOSIM *R* = 0.472, *P* < 0.05, Supplementary Figure S1D). Neither Shannon nor Simpson diversity index of the identified KEGG functions differed between the two cow groups (Supplementary Figure S2C and S2D). Among the 20 predominant pathways, 10 were significantly enriched (*P* < 0.05) in the HiEf cows than in the LoEf cows (Supplementary Figure S4A). A comparison of the abundances of all identified pathways between the two cow groups showed that a total of 34 of the 193 pathways were different (*P* < 0.05), with all being more abundant in the HiEf cows than in the LoEf cows (Supplementary Figure S4B). These pathways included carbon metabolism, glycolysis/gluconeogenesis, pyruvate metabolism, carbon fixation, purine metabolism, butanoate metabolism, oxidative phosphorylation, starch and sucrose metabolism, quorum sensing, and fructose and mannose metabolism. We further selected the key pathways involved in amino acid metabolism, carbohydrate metabolism, energy metabolism, and cofactors and vitamin metabolism. Four amino acid metabolism pathways, 11 carbohydrate metabolism pathways, two energy metabolism pathways, and two vitamin metabolism pathways were more abundant (*P* < 0.05) in the HiEf cows than in the LoEf cows (Fig. 3B).

The pathways detected using metatranscriptomics reflect the active metabolism, and they shall more closely reflect the actual functions at the time of sampling and host feed efficiency compared with the potential functions identified using metagenomics. We therefore further focused on the active functions only. The key enzymes involved in VFA and methane metabolism detected in the transcriptomes are presented in Supplementary Figure S5. A total of seven enzymes were significantly enriched in the HiEf cows (*P* < 0.05), four of which were involved in the succinate pathways and one of which was involved in the acrylate pathway of propionate formation, and the other two were involved in acetate and butyrate production. Three enzymes involved in the methanogenesis pathway were significantly enriched (*P* < 0.05) in the microbiome of the LoEf cows (Supplementary Figure S5).

### Potential microbial interactions identified in co-occurrence networks

**Fig. 4:**
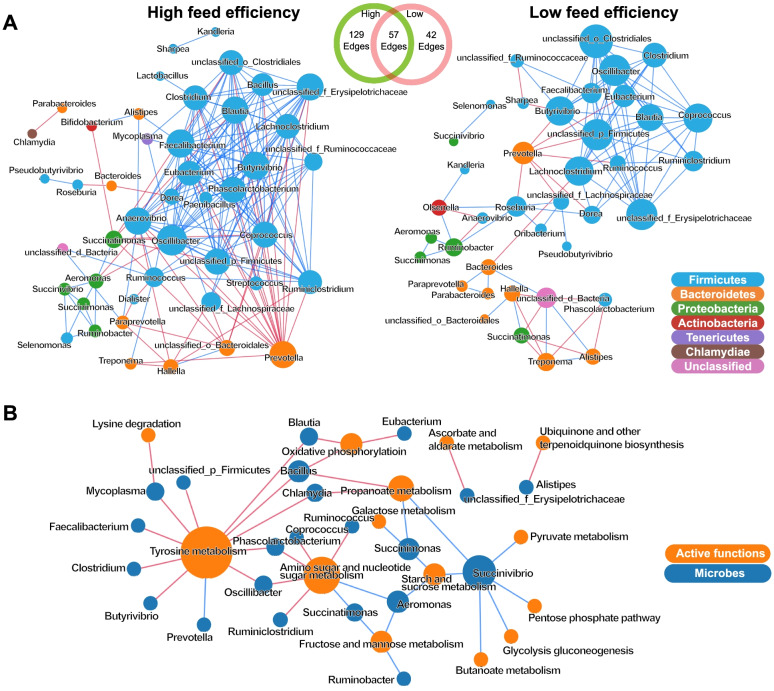
Co-occurrence networks of bacterial taxa. **A** The co-occurrence among rumen bacteria in the dairy cows with high and low feed efficiencies. **B** Relationships between rumen microbial taxa and feed efficiency-associated microbial functions. Only significant (*P* < 0.05) relationships are shown. Blue edges indicate positive relationships, and red edges indicate negative relationships. The node size is proportional to the mean abundance

Co-occurrence network analysis revealed a total of 228 co-occurrence relationships, with distinct co-occurrence patterns being found in each group of the cows with different feed efficiencies. In the rumen microbiome of HiEf animals, 186 connections were found, with the most positive relationships existing among taxa of *Firmicutes* and the most negative relationships existing between taxa of *Firmicutes* and taxa of *Bacteroidetes* (Fig. 4A). Notably, *Selenomonas*, which was significantly enriched in the HiEf animals, had positive relationships with *Succinivibrio*, *Succinimonas*, and *Ruminobacter.* These three taxa were positively correlated with *Aeromonas* and *Succinatimonas*. In the rumen microbiome of the LoEf animals, 99 relationships were observed, with taxa of *Firmicutes* positively correlated with each other but negatively correlated with *Prevotella* (Fig. 4A).

The co-occurrence network also revealed relationships between some rumen microbial taxa and functions associated with feed efficiency (Fig. 4B). A total of 16 positive and 24 negative correlations (*P* < 0.05) were found. Most of the feed efficiency-associated functions were those involved in carbohydrate metabolism and amino acid metabolism. The majority of the positive correlations (15 out of 16) were observed between *Succinivibrio*, *Succinimonas*, *Ruminobacter*, *Aeromonas*, or *Succinatimonas* and carbohydrate metabolism pathways (Fig. 4B).

### Microbe-metabolite interactions associated with host feed efficiency

**Fig. 5:**
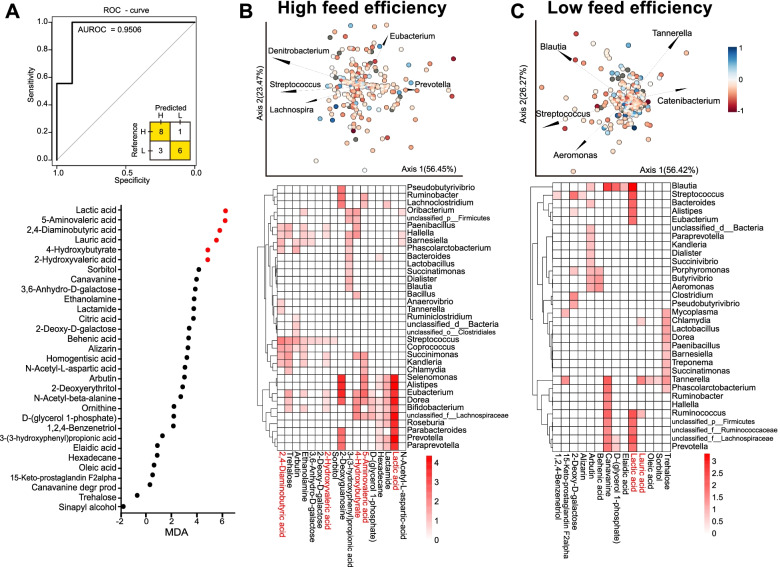
Prediction of host feed efficiency using rumen metabolites and microbe-metabolite interactions. Receiver operating characteristic (ROC) curve and the confusion matrix of the performance of the random forest model using the six selected metabolites (shown in red) whose mean decrease accuracy (MDA) was > 4 (**A**). Biplot drawn from the microbe-metabolite vectors (mmvec) conditional probabilities estimated for the dataset of high-efficiency (**B**) and low-efficiency (**C**) cows. Axes: principal components from the singular value decomposition of the microbe-metabolite conditional probabilities estimated using mmvec. Arrows: microbes, dots: metabolites, and colors of dots represent associations with host feed efficiency (blue: negative, red: positive). Heatmaps display the inferred conditional probabilities for various efficiency-associated metabolites given the presence of specific microbial taxa in the rumen of cows with high (**B**) and low (**C**) efficiencies

A total of 284 metabolites were identified in the rumen metabolomes (Supplementary Table S7). These metabolites were used in Spearman correlation analysis with the FCR phenotype to select the feed efficiency-associated metabolites. A total of 31 rumen metabolites were considered as efficiency-associated metabolites based on Spearman correlation (*P* < 0.05) and were used for predicting feed efficiency using the random forest model. Six of the feed efficiency-associated metabolites, including lactic acid, 5-aminovaleric acid, 2,4-diaminobutyric acid, lauric acid, 4-hydroxybutyrate, and 2-hydroxyvaleric acid, were selected by the random forest model, with an MDA > 4 (Fig. 5A). The receiver operating characteristic (ROC) curve represented an AUC of 0.9506 together with the inset confusion matrix. Of our cow cohort, 8 of the 9 HiEf animals were successfully predicted, and 6 of the 9 LoEf animals were successfully predicted. Cross-validation (99-fold) of the model achieved a Kappa coefficient of 0.71 and model error variance of 0.001.

To reveal meaningful relationships between genes and their products in the metabolomes, mmvec analysis was performed. Figure 5B and C show the different multi-omics biplots of microbe-metabolite interactions of HiEf and LoEf cows, respectively. Metabolites were colored according to their correlation coefficients with feed efficiency, showing no separation within metabolites in either efficient (Fig. 5B) or inefficient (Fig. 5C) animals. The heatmaps of the inferred conditional probabilities of specific metabolites indicated different interaction patterns of microbes and metabolites in the efficient vs. inefficient animals.

## Discussion

Animal performance of dairy cows, including feed efficiency, methane emissions, and milking traits, is largely determined or affected by the rumen microbiome [15, 16, 46]. To date, most previous studies have focused on the associations of rumen microbes with these host performance parameters using DNA-based analyses [2, 47, 48]. However, DNA-based analyses, including metagenomics, detect genes from both viable and dead microbes, and all genes irrespective of expression [49]. RNA-based analyses, including metatranscriptomics, overcome the above issues, though they also have some limitations, such as only detecting gene expression at the time of sample collection and not being able to address the biological challenge: RNA concentrations may not necessarily correlate with growth constantly [50]. Indeed, recent studies reported that different results could be obtained using metagenomics vs. metatranscriptomics [15, 17, 51]. A beef study concluded that transcriptionally active functions revealed by metatranscriptomics better reflects the actual active functions of microbiomes than the potential functions revealed by metagenomics [17]. In our study, metatranscriptomics identified half of the top 20 active pathways differing in abundance between dairy cows with high or low feed efficiency, while metagenomics identified none. Our study on dairy cattle together with previous studies on beef [17] and sheep [15, 51] indicate that metatranscriptomics is better suited to uncover the associations between rumen microbial functions and host performance. To maximize the discovery of such associations, we also used metabolomics. The integration of the three meta-omics helped address the question of how rumen microbes may function and interact to affect feed efficiency in dairy cows.

Comparison of the rumen microbial compositions between the two groups of cows revealed that bacteria and archaea, but not fungi, have significant associations with host feed efficiency. Most of the published studies focused on the rumen prokaryotes, emphasizing the importance of bacteria and archaea in affecting feed efficiency [8, 47, 52]. Rumen fungi play important roles in feed digestion [53, 54], but few studies have focused on their linkage to feed efficiency [9, 21]. Using metatranscriptomics, Zhang et al. [9] characterized the rumen eukaryotic community in beef cattle and found significant differences in protozoa and fungi among animals with different feed efficiencies [9]. The lack of difference in the fungal community between the two cow groups in our study contradicts the above study on beef cattle, but concur with a previous metagenomic study on dairy cattle [21]. Such discrepancies may be due to differences in animal species (beef vs. dairy) and methodologies (DNA- vs. RNA-based) used in different studies. Regarding archaea, the higher abundance of *Methanobrevibacter*, which is the most abundant methanogen in the rumen, in the LoEf animals is consistent with previous studies on beef [19] and dairy cattle [21]. More interestingly, the methanogens pathway was also upregulated in the metatranscriptome of the LoEf cows. These results suggest that more dietary energy might have been drained in the LoEf cows than in the HiEf cows. Breading for high-efficiency cows may also result in low-methane emitting cows.

Because bacteria play the most important role in feed digestion and fermentation, and utilization of the soluble monomers (sugar and amino acids) and secondary fermentation products (such as lactate, succinate, and H_2_) [55], their active metabolic functions and interactions with each other may determine or affect host feed efficiency. In the present study, the co-occurrence networks did reveal different occurrence patterns, which suggest potential interactions, in the bacterial communities between the two groups of cows with different feed efficiencies. The larger number of connections in the HiEf animals than in the LoEf animals (186 vs. 99) suggests more microbe-microbe interactions in the bacteriome of the former than the latter. The network of the HiEf cows included several genera of prevalent rumen bacteria in the hubs, including *Prevotella*, *Ruminiclostridium*, and *Oscillibacter* (MCC score > 100). These bacteria might play potential roles in the microbial interactions in the rumen microbiome of the HiEf cows.

The genus *Selenomonas* had a higher abundance in the rumen of the HiEf animals than of the LoEf cows, and the differential abundance was attributed to that of *Selenomonas bovis*, which is a species first isolated from the rumen of yak [56], but its metabolic functions in the rumen remain to be determined. The co-occurrence network analysis revealed that *Selenomonas* is positively correlated with several genera of the family *Succinivibrionaceae*, including *Ruminobacter*, *Succinivibrio*, and *Succinimonas*. Members of these genera have been reported to utilize sugars and some fermentation products of other microbes to produce succinate, lactate, acetate, and formate [55]. Most strains of *Selenomonas* can utilize starch producing primarily lactate, acetate, and propionate as end products [55, 57]. Members of *Succinivibrionaceae* primarily utilize hydrogen and produce succinate (a precursor of propionate), directing hydrogen away from methanogenesis [58]. Indeed, this taxon was found to be associated with high feed efficiency in cattle [52]. Taken the findings of the present study and previous studies together, *Selenomonas* and members of *Succinivibrionaceae* might have positively interacted with each other and played an important role as functional-keystone bacteria in the rumen of the HiEf animals owing to their ecological functions. The positive correlations between the abundances of these keystone members and their active metabolic functions further support their potential roles in the efficient utilization of feed in the HiEf animals.

In a previous study, we found that rumen metabolites had a stronger association with lactation performance (milk protein yield) in dairy cows than microbial compositions or functions [20]. Thus, in the present study, we also used metabolomics to explore the relationships between rumen microbial metabolites and feed efficiency. We found that six metabolites (mainly of carbohydrate metabolism) could predict host feed efficiency with an accuracy of 95.06%. Additionally, we found different microbe-metabolite interaction patterns in the rumen microbiome cows with different feed efficiency, and interestingly, these six markers were important contributors and played key roles in differentiating these microbe-metabolite interactions between the high- and low-efficiency cows. The development of machine learning methods facilitates the application of the microbiota to predict host phenotypes, including for the prediction of disease risk [59] and performance [60] in different animal species. However, to our knowledge, research is still limited on the prediction of host performance based on rumen microbial metabolites, especially in ruminants. Notably, predicting phenotypes using omics data in practice is still challenging due to the different characterization methods used for the identification of microbial or metabolic markers [61]. Therefore, further studies using standardized omics identification pipelines are required to test the robustness of these markers, which could help us apply our findings in practice in the near future.

Studies using multiple meta-omics are still costly, and thus, the several recent studies using multiple meta-omics on ruminants all had a small sample size (3-10) [17, 51, 62]. The sample size (9 per cow group) of the present study was larger than those reported in similar studies, but still relatively small. As stated in the “Methods,” our sample size provided sufficient power with respect to animal phenotypic data (statistical power: 99.67%, effect size: 0.78). The ANOSIM of the metagenomic and metatranscriptomic data indicate that the differences between the two cow groups were significantly larger than the variations among individual animals in each group. Nevertheless, a larger sample size should be considered in future multi-omics studies aiming at investigating the microbial mechanisms contributing to host performances.

**Fig. 6:**
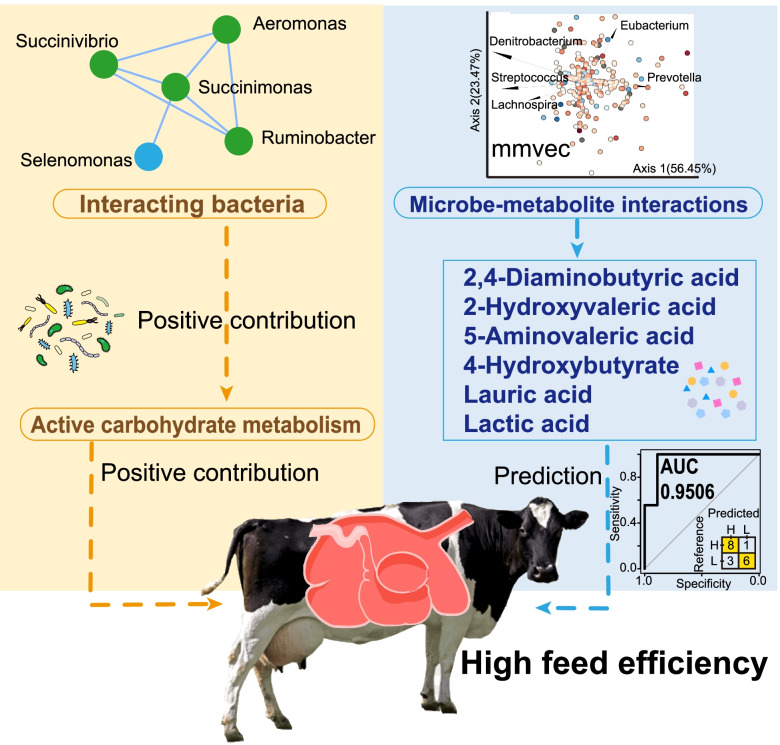
A working model to illustrate the microbial taxa, active carbohydrate metabolism, and metabolites that might be associated with feed efficiency in dairy cows

Taken together, integrating metagenomics, metatranscriptomics, and metabolomics, we uncovered some new features of the rumen microbiome that are potentially associated with feed efficiency in dairy cows (Fig. 6). The new insights into these rumen microbial taxonomic, functional, and metabolic features may improve the ability to select animals for better performance in the dairy industry. The fundamental knowledge will also inform future interventions to improve feed efficiency in dairy cows.

## Conclusions

Compared to metagenomics, metatranscriptomics is a better approach to uncover the associations between rumen microbial functions and feed efficiency in dairy cows. Co-occurrence analysis revealed that differential interaction patterns might exist in the rumen microbiomes of animals with different feed efficiencies and that the microbiomes of the HiEf animals have stronger associations. Notably, in the rumen of HiEf animals, *Selenomonas* and some species of *Succinivibrionaceae* might interact positively with each other and play an important role as keystone bacteria. The six metabolites (all derived from carbohydrate metabolism) identified by random forest analysis could potentially serve as metabolic markers to differentiate efficient and inefficient rumen microbiomes or dairy cows. The findings of the present study may help future research to breed or select high-efficiency cows and inform sourcing carbohydrate-degrading enzymes from the rumen as feed additives or biocatalysts to harness bioenergy from lignocellulosic biomass.

## Supplementary Information

**Additional file 1: Supplementary Table S1.** Comparison of physiological parameters between low- and high-efficiency cows. **Supplementary Table S2.** Summary of metagenomic and metatranscriptomic sequencing data. **Supplementary Table S3.** Predominant phyla, genera, and species of bacteria, archaea, and fungi identified in the metagenomes. **Supplementary Table S4.** Predominant phyla, genera, and species of bacteria, archaea, and fungi identified in the metatranscriptomes. **Supplementary Table S5.** KEGG level-3 pathways identified in the metagenomes. **Supplementary Table S6.** KEGG level-3 pathways identified in the metatranscriptomes. **Supplementary Table S7.** Metabolites identified in the rumen metabolomes of the 18 dairy cattle.

**Additional file 2: Supplementary Figure S1.** Non-metric multi-dimensional scaling (NMDS) analysis based on Bray-Curtis dissimilarity of microbial species (A and B) and functions (C and D) calculated from the metagenomic data and metatranscriptomic data, respectively.

**Additional file 3: Supplementary Figure S2.** Shannon diversity index and Simpson diversity index of species (A and B) and functions (C and D) calculated from the metatranscriptomic data. The Wilcoxon rank-sum test was used for mean comparison. *, *P* < 0.05.

**Additional file 4: Supplementary Figure S3.** The 20 most abundant KEGG pathways identified in the metagenomes of the two cow groups (A) and fold changes (HiEf/LoEf) of the KEGG pathways that significantly differed between the two cow groups (B).

**Additional file 5:** **Supplementary Figure S4.** The 20 most abundant KEGG pathways identified in the metatranscriptomes of the two cow groups (A), and fold changes (HiEf/LoEf) of the KEGG pathways that significantly differed between the two cow groups (B). The Wilcoxon rank-sum test was used for mean comparison. *, *P* < 0.05.

**Additional file 6:** **Supplementary Figure S5.** Metabolic pathways of VFA production and hydrogenotrophic methanogenesis in which differentially regulated genes were found between the two cow groups. Blue: genes significantly upregulated in the rumen of high-efficiency cows. Red: genes significantly upregulated in the rumen of low-efficiency cows.

## Data Availability

Sequencing data were deposited into the NCBI Sequence Read Archive (SRA) under the accession number of PRJNA597489.

## References

[1] Kearney J (2010). Food consumption trends and drivers. Philos Trans Roy Soc Lond B Biol Sci.

[2] Mizrahi I, Jami E (2018). Review: the compositional variation of the rumen microbiome and its effect on host performance and methane emission. Animal..

[3] Løvendahl P, Difford GF, Li B, Chagunda MGG, Huhtanen P, Lidauer MH (2018). Review: selecting for improved feed efficiency and reduced methane emissions in dairy cattle. Animal..

[4] Huhtanen P, Cabezas-Garcia EH, Utsumi S, Zimmerman S (2015). Comparison of methods to determine methane emissions from dairy cows in farm conditions. J Dairy Sci.

[5] Cabezas-Garcia EH, Krizsan SJ, Shingfield KJ, Huhtanen P (2017). Between-cow variation in digestion and rumen fermentation variables associated with methane production. J Dairy Sci.

[6] Guan LL, Nkrumah JD, Basarab JA, Moore SS (2008). Linkage of microbial ecology to phenotype: correlation of rumen microbial ecology to cattle's feed efficiency. FEMS Microbiol Lett.

[7] Carberry CA, Kenny DA, Han S, Mccabe MS, Waters SM (2012). Effect of phenotypic residual feed intake and dietary forage content on the rumen microbial community of beef cattle. Appl Environ Microbiol.

[8] Jewell KA, McCormick CA, Odt CL, Weimer PJ, Suen G (2015). Ruminal bacterial community composition in dairy cows is dynamic over the course of two lactations and correlates with feed efficiency. Appl Environ Microbiol.

[9] Zhang YW, Li FY, Chen YH, Wu H, Meng QX, Guan LL (2020). Metatranscriptomic profiling reveals the effect of breed on active rumen eukaryotic composition in beef cattle with varied feed efficiency. Front Microbiol.

[10] Bergman EN (1990). Energy contributions of volatile fatty acids from the gastrointestinal tract in various species. Physiol Rev.

[11] Kay BRN (1969). Digestion of protein in the intestines of adult ruminants. Proc Nutr Soc.

[12] Hernandez-Sanabria E, Guan LL, Goonewardene LA, Li M, Mujibi DF, Stothard P (2010). Correlation of particular bacterial PCR-denaturing gradient gel electrophoresis patterns with bovine ruminal fermentation parameters and feed efficiency traits. Appl Environ Microbiol.

[13] Carberry CA, Waters SM, Kenny DA, Creevey CJ (2014). Rumen methanogenic genotypes differ in abundance according to host residual feed intake phenotype and diet type. Appl Environ Microbiol.

[14] McCann JC, Wiley LM, Forbes TD, Rouquette FM, Tedeschi LO (2014). Relationship between the rumen microbiome and residual feed intake-efficiency of Brahman bulls stocked on bermudagrass pastures. PLoS One.

[15] Shi W, Moon CD, Leahy SC, Kang D, Froula J, Kittelmann S (2014). Methane yield phenotypes linked to differential gene expression in the sheep rumen microbiome. Genome Res.

[16] Roehe R, Dewhurst RJ, Duthie CA, Rooke JA, Mckain N, Ross DW (2016). Bovine host genetic variation influences rumen microbial methane production with best selection criterion for low methane emitting and efficiently feed converting hosts based on metagenomic gene abundance. PLoS Genet.

[17] Li FY, Hitch TCA, Chen YH, Creevey CJ, Guan LL (2019). Comparative metagenomic and metatranscriptomic analyses reveal the breed effect on the rumen microbiome and its associations with feed efficiency in beef cattle. Microbiome..

[18] Taxis TM, Wolff S, Gregg SJ, Minton NO, Zhang C, Dai J (2015). The players may change but the game remains: network analyses of ruminal microbiomes suggest taxonomic differences mask functional similarity. Nucleic Acids Res.

[19] Li F, Guan Y, LL. (2017). Metatranscriptomic profiling reveals linkages between the active rumen microbiome and feed efficiency in beef cattle. Appl Environ Microbiol.

[20] Xue MY, Sun HZ, Wu XH, Liu JX, Guan LL (2020). Multi-omics reveals that the rumen microbiome and its metabolome together with the host metabolome contribute to individualized dairy cow performance. Microbiome..

[21] Shabat SKB, Sasson G, Doronfaigenboim A, Durman T, Yaacoby S, Miller MEB, et al. Specific microbiome-dependent mechanisms underlie the energy harvest efficiency of ruminants. ISME J. 2016;10:2958–72. 10.1038/ismej.2016.62 PMC5148187 27152936

[22] Xie YY, Wu ZZ, Wang DM, Liu J (2019). Nitrogen partitioning and microbial protein synthesis in lactating dairy cows with different phenotypic residual feed intake. J Anim Sci Biotechnol.

[23] Shen JS, Chai Z, Song LJ, Liu JX, Wu YM (2012). Insertion depth of oral stomach tubes may affect the fermentation parameters of ruminal fluid collected in dairy cows. J Dairy Sci.

[24] Yu Z, Morrison M (2004). Improved extraction of PCR-quality community DNA from digesta and fecal samples. BioTechniques..

[25] Li H, Durbin R (2009). Fast and accurate short read alignment with Burrows–Wheeler transform. Bioinformatics..

[26] Li D, Liu CM, Luo R, Sadakane K, Lam T-W (2015). MEGAHIT: an ultra-fast single-node solution for large and complex metagenomics assembly via succinct de Bruijn graph. Bioinformatics..

[27] Noguchi H, Park J, Takagi T (2006). MetaGene: prokaryotic gene finding from environmental genome shotgun sequences. Nucleic Acids Res.

[28] Fu LM, Niu BF, Zhu WZ, Wu ST, Li WZ (2012). CD-HIT: accelerated for clustering the next-generation sequencing data. Bioinformatics..

[29] Yu C, Wang J, Kristiansen K, Li R, Yiu S-M, Lam T-W (2009). SOAP2: an improved ultrafast tool for short read alignment. Bioinformatics..

[30] Buchfink B, Xie C, Huson DH (2014). Fast and sensitive protein alignment using DIAMOND. Nat Methods.

[31] Pruitt KD, Tatusova T, Maglott DR (2006). NCBI reference sequences (RefSeq): a curated non-redundant sequence database of genomes, transcripts and proteins. Nucleic Acids Res.

[32] Li F, Henderson G, Sun X, Cox F, Janssen PH, Guan LL (2016). Taxonomic assessment of rumen microbiota using total RNA and targeted amplicon sequencing approaches. Front Microbiol.

[33] Poulsen M, Schwab C, Borg Jensen B, Engberg RM, Spang A, Canibe N (2013). Methylotrophic methanogenic Thermoplasmata implicated in reduced methane emissions from bovine rumen. Nat Commun.

[34] Bolger AM, Lohse M, Usadel B (2014). Trimmomatic: a flexible trimmer for Illumina sequence data. Bioinformatics..

[35] Kim D, Pertea G, Trapnell C, Pimentel H, Kelley R, Salzberg SL (2013). TopHat2: accurate alignment of transcriptomes in the presence of insertions, deletions and gene fusions. Genome Biol.

[36] Namiki T, Hachiya T, Tanaka H, Sakakibara Y (2012). MetaVelvet: an extension of Velvet assembler to de novo metagenome assembly from short sequence reads. Nucleic Acids Res.

[37] Edgar RC (2010). Search and clustering orders of magnitude faster than BLAST. Bioinformatics..

[38] Meyer F, Paarmann D, D'Souza M, Olson R, Glass EM, Kubal M (2008). The metagenomics RAST server – a public resource for the automatic phylogenetic and functional analysis of metagenomes. BMC Bioinformatics.

[39] Westreich ST, Korf I, Mills DA, Lemay DG (2016). SAMSA: a comprehensive metatranscriptome analysis pipeline. BMC Bioinformatics.

[40] Kind T, Wohlgemuth G, Lee DY, Lu Y, Palazoglu M, Shahbaz S (2009). FiehnLib: mass spectral and retention index libraries for metabolomics based on quadrupole and time-of-flight gas chromatography/mass spectrometry. Anal Chem.

[41] Friedman J, Alm EJ (2012). Inferring correlation networks from genomic survey data. PLoS Comput Biol.

[42] Breiman L (2001). Random Forests. Mach Learn.

[43] Morton JT, Aksenov AA, Nothias LF, Foulds JR, Quinn RA, Badri MH (2019). Learning representations of microbe–metabolite interactions. Nat Methods.

[44] Bolyen E, Rideout JR, Dillon MR, Bokulich NA, Abnet CC, Al-Ghalith GA (2019). Reproducible, interactive, scalable and extensible microbiome data science using QIIME 2. Nat Biotechnol.

[45] Benjamini Y, Hochberg Y (1995). Controlling the false discovery rate - a practical and powerful approach to multiple testing. J Roy Stat Soc B.

[46] Jami E, Mizrahi I (2012). Composition and similarity of bovine rumen microbiota across individual animals. PLoS One.

[47] Myer PR, Smith TP, Wells JE, Kuehn LA, Freetly HC (2015). Rumen microbiome from steers differing in feed efficiency. PLoS One.

[48] Jami E, White BA, Mizrahi I (2014). Potential role of the bovine rumen microbiome in modulating milk composition and feed efficiency. PLoS One.

[49] Denman SE, Morgavi DP, McSweeney CS (2018). Review: the application of omics to rumen microbiota function. Animal..

[50] Blazewicz SJ, Barnard RL, Daly RA, Firestone MK (2013). Evaluating rRNA as an indicator of microbial activity in environmental communities: limitations and uses. ISME J.

[51] Kamke J, Kittelmann S, Soni P, Li Y, Tavendale M, Ganesh S (2016). Rumen metagenome and metatranscriptome analyses of low methane yield sheep reveals a Sharpea-enriched microbiome characterised by lactic acid formation and utilisation. Microbiome..

[52] Li FY, Li CX, Chen YH, Liu JH, Zhang CY, Irving B (2019). Host genetics influence the rumen microbiota and heritable rumen microbial features associate with feed efficiency in cattle. Microbiome..

[53] Gruninger RJ, Puniya AK, Callaghan TM, Edwards JE, Youssef N, Dagar SS (2014). Anaerobic fungi (phylum Neocallimastigomycota): advances in understanding their taxonomy, life cycle, ecology, role and biotechnological potential. FEMS Microbiol Ecol.

[54] Henderson G, Cox F, Kittelmann S, Miri VH, Zethof M, Noel SJ (2013). Effect of DNA extraction methods and sampling techniques on the apparent structure of cow and sheep rumen microbial communities. PLoS One.

[55] Mizrahi I, Wallace RJ, Moraïs S. The rumen microbiome: balancing food security and environmental impacts. Nat Rev Microbiol. 2021;19:553–66. 10.1038/s41579-021-00543-6 33981031

[56] Zhang KG, Dong XZ (2009). Selenomonas bovis sp. nov., isolated from yak rumen contents. Int J Syst Evol Microbiol.

[57] Dehority BA, Grubb JA (1977). Characterization of the predominant bacteria occurring in the rumen of goats (Capra hircus). Appl Environ Microbiol.

[58] Pope PB, Smith W, Denman SE, Tringe SG, Barry K, Hugenholtz P (2011). Isolation of Succinivibrionaceae implicated in low methane emissions from Tammar wallabies. Science..

[59] Ma T, Villot C, Renaud D, Skidmore A, Chevaux E, Steele M (2020). Linking perturbations to temporal changes in diversity, stability, and compositions of neonatal calf gut microbiota: prediction of diarrhea. ISME J.

[60] Wang XF, Tsai TC, Deng FL, Wei XY, Chai JM, Knapp J (2019). Longitudinal investigation of the swine gut microbiome from birth to market reveals stage and growth performance associated bacteria. Microbiome..

[61] Gloor GB, Wu JR, Pawlowsky-Glahn V, Egozcue JJ (2016). It's all relative: analyzing microbiome data as compositions. Ann Epidemiol.

[62] Shen H, Lu ZY, Xu ZH, Chen Z, Shen ZM (2017). Associations among dietary non-fiber carbohydrate, ruminal microbiota and epithelium G-protein-coupled receptor, and histone deacetylase regulations in goats. Microbiome..

