# Supplementary figures and images for: Integrated meta-omics reveals new ruminal microbial features associated with feed efficiency in dairy cattle
Source: Microbiome. 2022 Feb 16;10:32. doi: 10.1186/s40168-022-01228-9 (PMC8849036; doi:10.1186/s40168-022-01228-9)

# Microbial NMDS

**A**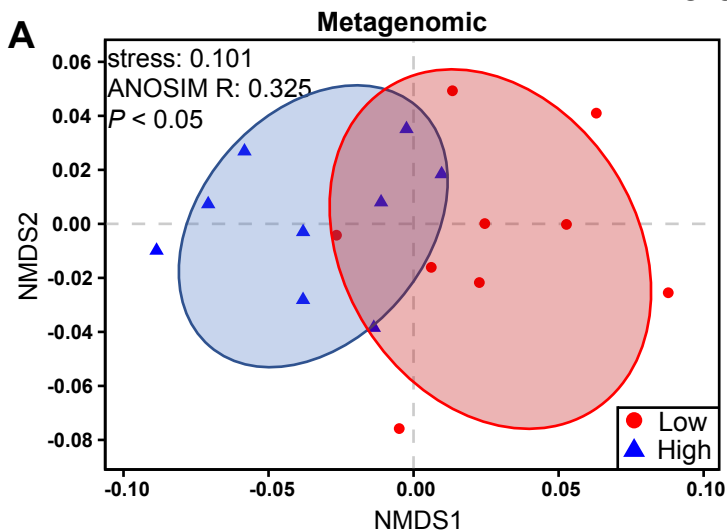**B**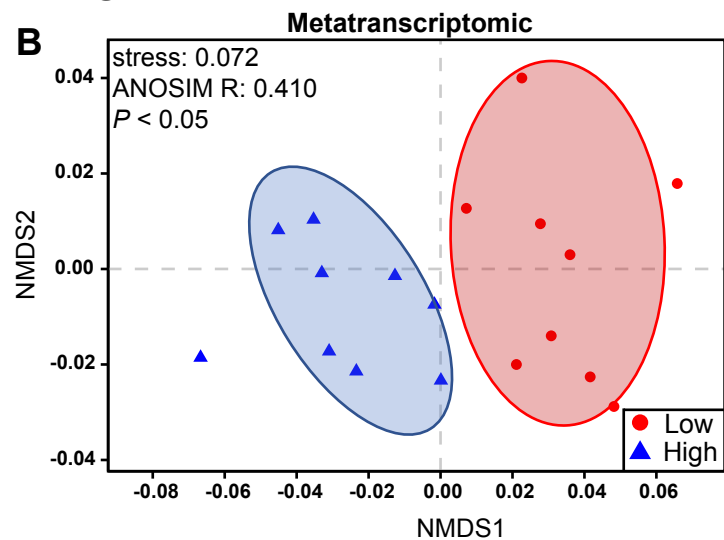

# Functional NMDS

**C**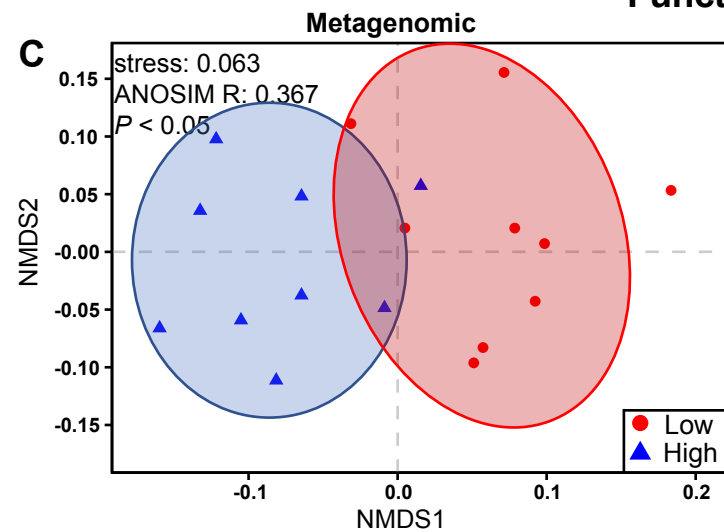**D**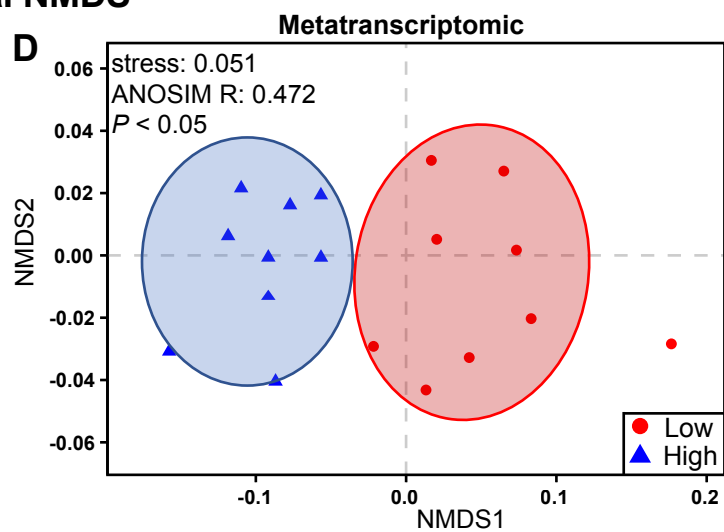

Supplement: Supplementary file 3 — Additional file 2: Supplementary Figure S1. Non-metric multi-dimensional scaling (NMDS) analysis based on Bray-Curtis dissimilarity of microbial species (A and B) and functions (C and D) calculated from the metagenomic data and metatranscriptomic data, respectively. [file 40168_2022_1228_MOESM3_ESM.pdf]

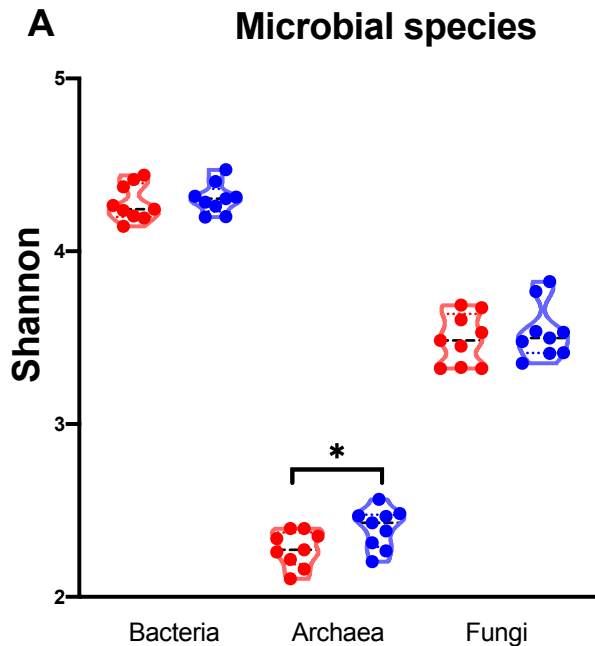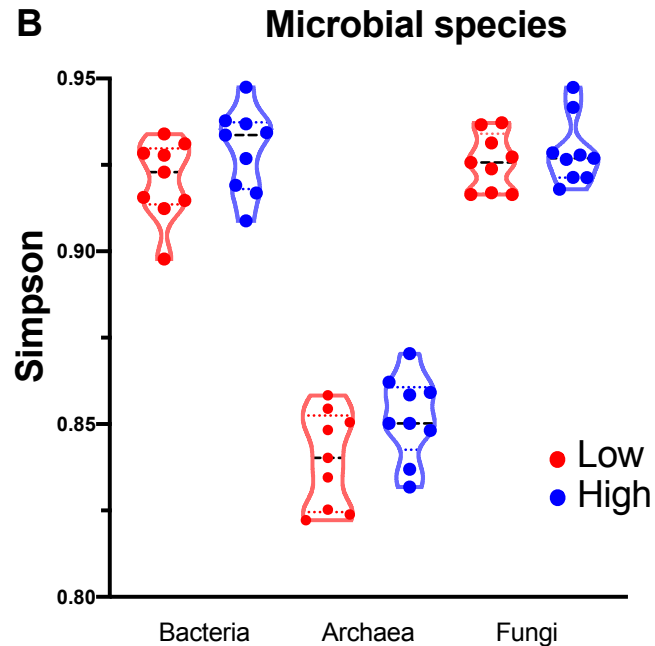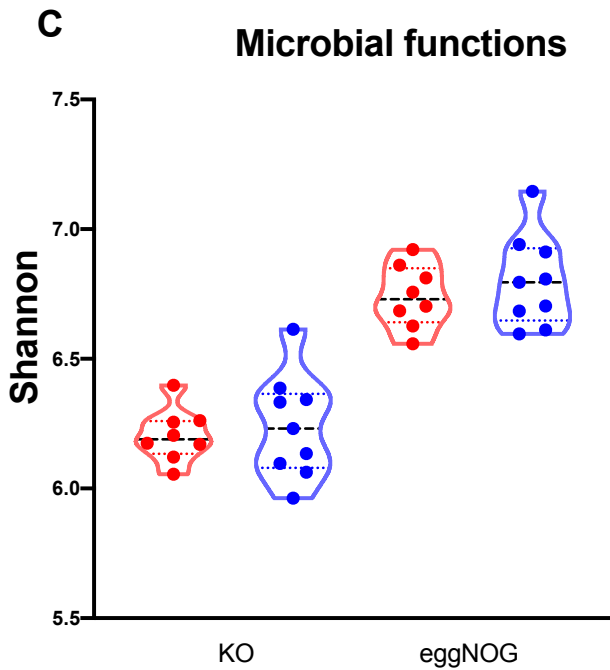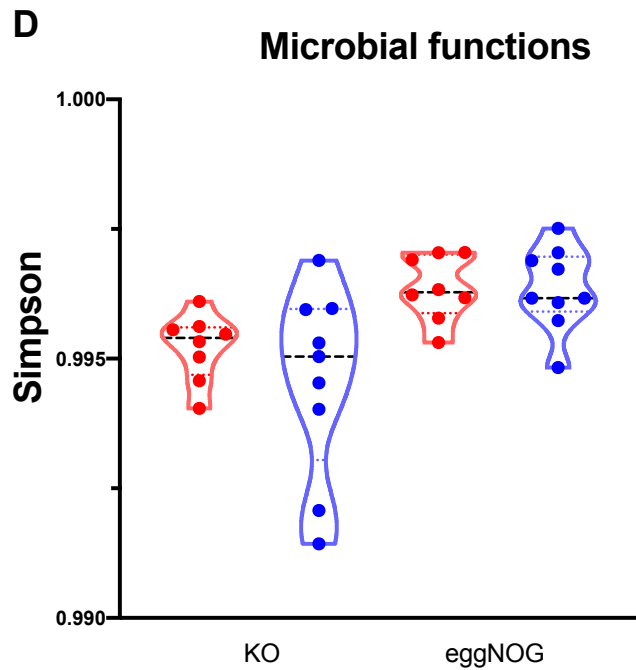

Supplement: Supplementary file 4 — Additional file 3: Supplementary Figure S2. Shannon diversity index and Simpson diversity index of species (A and B) and functions (C and D) calculated from the metatranscriptomic data. The Wilcoxon rank-sum test was used for mean comparison. *, P < 0.05. [file 40168_2022_1228_MOESM4_ESM.pdf]

**A**

Top 20 metagenomic KEGG pathways

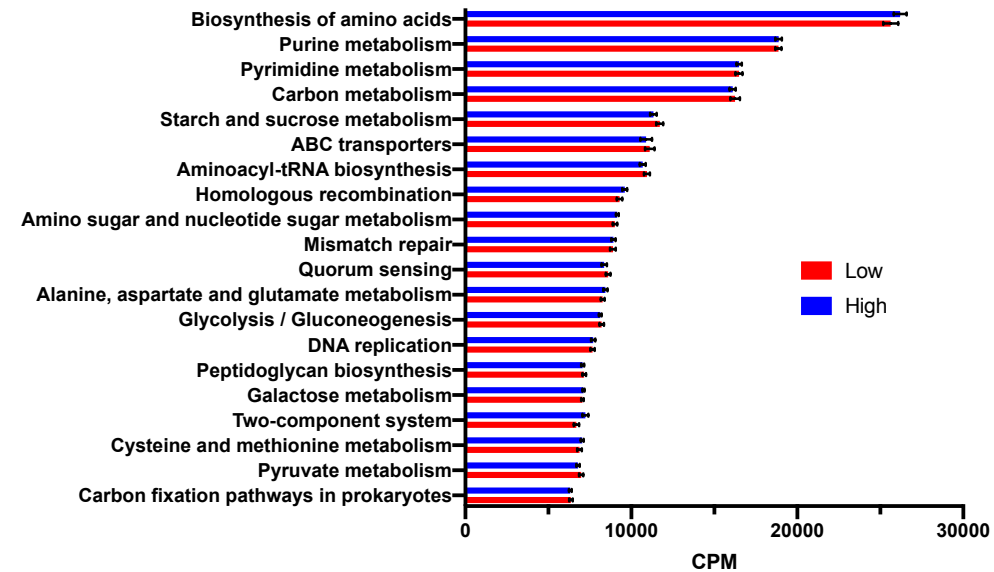**B**

Significantly different metagenomic KEGG pathways

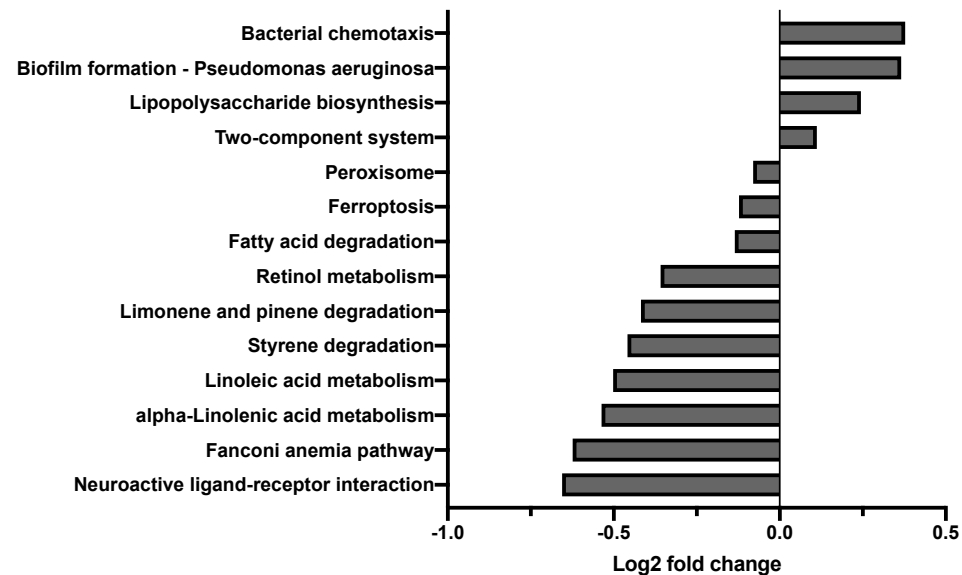

Supplement: Supplementary file 5 — Additional file 4: Supplementary Figure S3. The 20 most abundant KEGG pathways identified in the metagenomes of the two cow groups (A) and fold changes (HiEf/LoEf) of the KEGG pathways that significantly differed between the two cow groups (B). [file 40168_2022_1228_MOESM5_ESM.pdf]

# Pyruvate

■ Enriched in high efficiency cows

■ Enriched in low efficiency cows

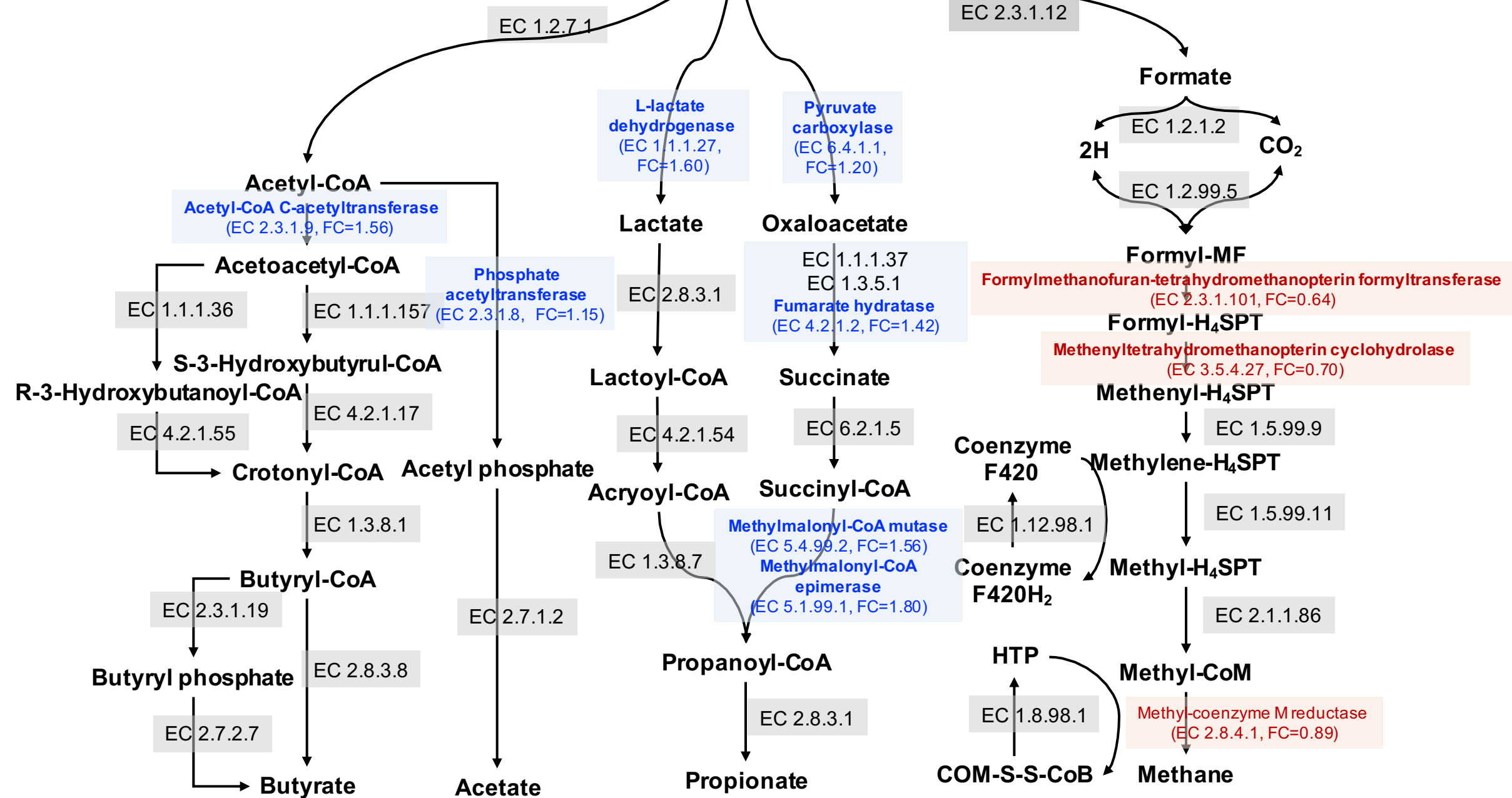

Supplement: Supplementary file 7 — Additional file 6: Supplementary Figure S5. Metabolic pathways of VFA production and hydrogenotrophic methanogenesis in which differentially regulated genes were found between the two cow groups. Blue: genes significantly upregulated in the rumen of high-efficiency cows. Red: genes significantly upregulated in the rumen of low-efficiency cows. [file 40168_2022_1228_MOESM7_ESM.pdf]
