# Supplementary material for: Integrated meta-omics reveals new ruminal microbial features associated with feed efficiency in dairy cattle
Source: Microbiome. 2022 Feb 16;10:32. doi: 10.1186/s40168-022-01228-9 (PMC8849036; doi:10.1186/s40168-022-01228-9)
Supplement: Supplementary file 6 — Additional file 5: Supplementary Figure S4. The 20 most abundant KEGG pathways identified in the metatranscriptomes of the two cow groups (A), and fold changes (HiEf/LoEf) of the KEGG pathways that significantly differed between the two cow groups (B). The Wilcoxon rank-sum test was used for mean comparison. *, P < 0.05. [file 40168_2022_1228_MOESM6_ESM.pdf]

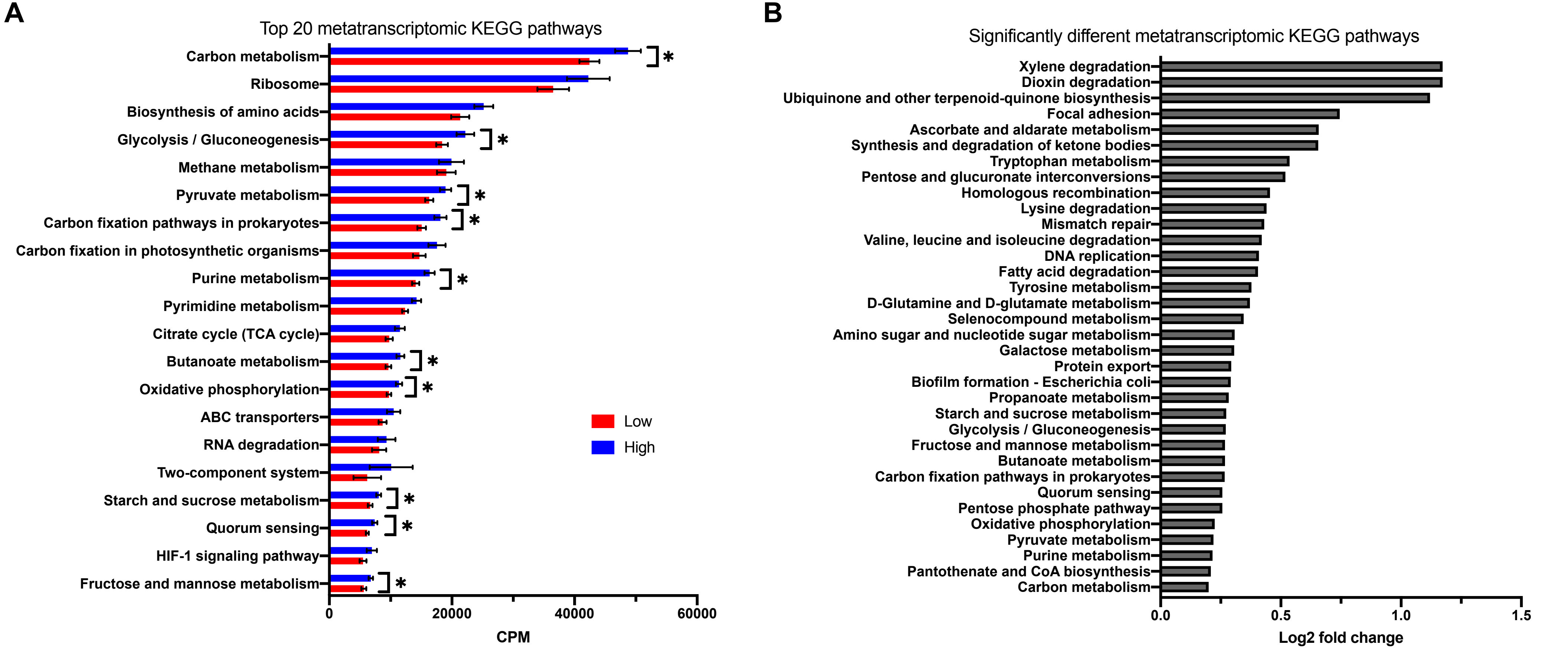

A
Top 20 metatranscriptomic KEGG pathways
Carbon metabolism
Ribosome
Biosynthesis of amino acids
Glycolysis / Gluconeogenesis
Methane metabolism
Pyruvate metabolism
Carbon fixation pathways in prokaryotes
Carbon fixation in photosynthetic organisms
Purine metabolism
Pyrimidine metabolism
Citrate cycle (TCA cycle)
Butanoate metabolism
Oxidative phosphorylation
ABC transporters
RNA degradation
Two-component system
Starch and sucrose metabolism
Quorum sensing
HIF-1 signaling pathway
Fructose and mannose metabolism
Low
High
0
20000
40000
60000
CPM
B
Significantly different metatranscriptomic KEGG pathways
Xylene degradation
Dioxin degradation
Ubiquinone and other terpenoid-quinone biosynthesis
Focal adhesion
Ascorbate and aldarate metabolism
Synthesis and degradation of ketone bodies
Tryptophan metabolism
Pentose and glucuronate interconversions
Homologous recombination
Lysine degradation
Mismatch repair
Valine, leucine and isoleucine degradation
DNA replication
Fatty acid degradation
Tyrosine metabolism
D-Glutamine and D-glutamate metabolism
Selenocompound metabolism
Amino sugar and nucleotide sugar metabolism
Galactose metabolism
Protein export
Biofilm formation - Escherichia coli
Propanoate metabolism
Starch and sucrose metabolism
Glycolysis / Gluconeogenesis
Fructose and mannose metabolism
Butanoate metabolism
Carbon fixation pathways in prokaryotes
Quorum sensing
Pentose phosphate pathway
Oxidative phosphorylation
Pyruvate metabolism
Purine metabolism
Pantothenate and CoA biosynthesis
Carbon metabolism
0.0
0.5
1.0
1.5
Log2 fold change
